# Lessons learned from implementing a surge capacity support program for COVID-19 contact management in Ontario

**DOI:** 10.17269/s41997-023-00773-6

**Published:** 2023-05-03

**Authors:** Andrea Chambers, Jacquelyn Quirk, Elaina A. MacIntyre, Andrea Bodkin, Heather Hanson

**Affiliations:** 1 Public Health Ontario, 480 University Avenue, Suite 300, Toronto, ON M5G 1V2 Canada; 2 University of Toronto, Toronto, ON Canada

**Keywords:** COVID-19, Case and contact management, Contact tracing, Surge capacity, Public health, Evaluation, COVID-19, gestion des cas et des contacts, recherche des contacts, capacité de mobilisation, santé publique, évaluation

## Abstract

**Setting:**

In Ontario, local public health units (PHUs) are responsible for leading case investigations, contact tracing, and follow-up. The workforce capacity and operational requirements needed to maintain this public health strategy during the COVID-19 pandemic were unprecedented.

**Intervention:**

Public Health Ontario’s Contact Tracing Initiative (CTI) was established to provide a centralized workforce. This program was unique in leveraging existing human resources from federal and provincial government agencies and its targeted focus on initial and follow-up phone calls to high-risk close contacts of COVID-19 cases. By setting criteria for submissions to the program, standardizing scripts, and simplifying the data management process, the CTI was able to support a high volume of calls.

**Outcomes:**

During its 23 months of operation, the CTI was used by 33 of the 34 PHUs and supported over a million calls to high-risk close contacts. This initiative was able to meet its objectives while adapting to the changing dynamics of the pandemic and the implementation of a new COVID-19 provincial information system. Core strengths of the CTI were timeliness, volume, and efficient use of resources. The CTI was found to be useful for school exposures, providing support when public health measures were lifted, and in supporting PHU’s reallocation of resources during the vaccine roll-out.

**Implications:**

When considering future use of this model, it is important to take note of the program strengths and limitations to ensure alignment with future needs for surge capacity support. Lessons learned from this initiative could provide practice-relevant knowledge for surge capacity planning.

**Supplementary Information:**

The online version contains supplementary material available at 10.17269/s41997-023-00773-6.

## Résumé

**Contexte:**

En Ontario, ce sont les bureaux de santé publique qui s’occupent des enquêtes de cas, de la recherche des contacts et des suivis. Pendant la pandémie de COVID-19, les besoins opérationnels et de capacité de la main-d’œuvre à combler pour conserver cette stratégie de santé publique ont atteint une ampleur jamais vue.

**Intervention:**

L’Initiative de recherche des contacts dans le cadre de la lutte contre la COVID-19 de Santé publique Ontario a été mise sur pied dans l’objectif de centraliser l’effectif. Mobilisant des ressources humaines d’organisations fédérales et provinciales, ce programme a permis de faire les appels initiaux et de suivi aux contacts étroits de cas de COVID-19 exposés à un risque élevé. Grâce à des critères bien établis pour les soumissions au programme, à l’uniformisation des scripts et à la simplification du processus de gestion des données, un grand volume d’appels a pu être traité.

**Résultats:**

Durant les 23 mois de l’Initiative, 33 des 34 bureaux de santé publique y ont eu recours. Ce sont ainsi plus d’un million d’appels à des contacts étroits qui ont pu être faits. L’Initiative a permis d’atteindre les objectifs en s’adaptant au contexte pandémique en constante évolution et de mettre en œuvre un nouveau système de gestion des renseignements provinciaux sur la COVID-19. Ses grandes forces sont la rapidité, le volume et l’efficacité de l’utilisation des ressources. Elle a été particulièrement utile dans les cas d’exposition en milieu scolaire, permettant d’offrir du soutien à la levée des mesures sanitaires et d’aider à la réaffectation des ressources des bureaux de santé publique pendant la campagne de vaccination.

**Conséquences:**

Si l’on envisage de réutiliser ce modèle, il importe de tenir compte des forces et des faiblesses du programme pour qu’il cadre avec les besoins futurs de soutien en matière de capacité de mobilisation. Les leçons tirées de cette initiative pourraient s’avérer pertinentes pour la planification de cette capacité.

## Introduction

In the early days of the COVID-19 pandemic, case and contact management was identified as an important strategy for interrupting chains of transmission and reducing mortality associated with COVID-19 (WHO, 2021). Case investigations are designed to notify individuals with suspected or confirmed infection and identify individuals who may be considered high risk based on their exposure. Contact tracing involves locating, notifying, and interviewing high-risk close contacts of suspected or confirmed cases. Information can be shared to help contacts understand their risk of exposure, how to prevent further transmission, and access health care services.

Public Health Ontario’s (PHO) Contact Tracing Initiative (CTI) was established in Ontario to provide a centralized workforce leveraging existing human resources from federal and provincial agencies. The CTI was focused solely on contact notification; that is, providing initial and follow-up phone calls to high-risk close contacts of confirmed or probable cases of COVID-19. The CTI was designed to support a high volume of calls and be responsive to shifts in the need for surge capacity support.

This paper describes how this program was designed and implemented, how it was used by public health units (PHUs), and overall lessons learned that could inform future models to increase capacity for contact follow-up support.

## Setting

In Ontario, 34 regional PHUs are responsible for leading case and contact management for their respective regions. There is a duty to report *Diseases of Public Health Significance*, as defined by the Ministry of Health (MOH), including COVID-19. Case definitions for all *Diseases of Public Health Significance* and the direction for their public health management are established by the MOH. During the COVID-19 pandemic, the MOH routinely updated provincial requirements for case and contact management and monitored workforce capacity at the PHU level. PHO is a partner in Ontario’s public health system, delivering public health laboratory services and providing scientific and technical advice to support public health practice.

## Intervention

### Overview

In mid-March 2020, the MOH requested that PHO develop a centralized program to support Ontario’s 34 PHUs with contact management. The MOH was meeting routinely with PHUs to assess capacity across a number of areas. It was quickly recognized that some PHUs would reach capacity limits to maintain provincial requirements for case and contact follow-up. PHO was asked to explore options for providing support based on available resources. The workforce of several provincial and federal agencies was offered to make daily contact follow-up calls. There were no existing protocols in place to define roles, responsibilities, and processes for a provincial contract tracing program.

Surge capacity has been described as the ability of a facility or system to expand its operations to manage an influx of patients in response to an incident (Bonnett et al., 2007). Bonnett et al. presented a foundational conceptual framework for understanding surge capacity, describing facility-based surge capacity as the step where facilities modify their operations to manage an increase in volume and extrinsic surge capacity as the step where facilities need to transition to receiving outside help.

**Fig. 1:**
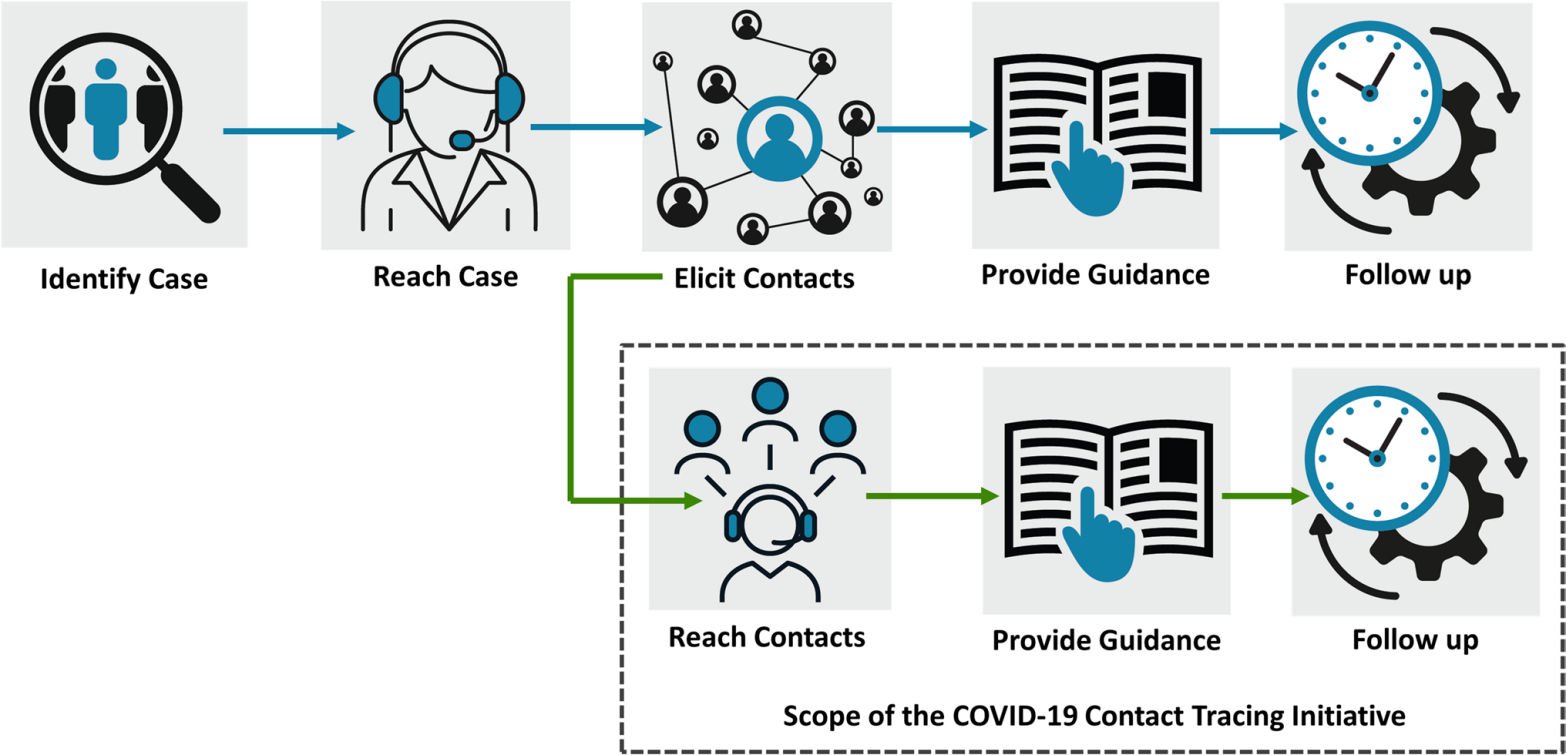
Overview of the case and contact management process and where Public Health Ontario’s Contact Tracing Initiative provided surge capacity support

The CTI was developed as a centralized program that provided PHUs with extrinsic surge capacity support. It was designed to support PHUs’ provincial requirements to notify and follow up with high-risk close contacts of COVID-19 cases. Figure 1 highlights what tasks in the case and contact management process were supported by the CTI. Phone calls to high-risk close contacts involved a symptom assessment and the delivery of information about testing and self-isolation. Translation services were available to provide simultaneous interpretation in French and a variety of other languages.

### Partnerships

PHO was responsible for developing the CTI and providing ongoing support for operations. Public health physicians, nurse consultants, and adult education specialists developed call processes, scripts, and training resources and provided ongoing support. PHO was also able to leverage existing internal expertise around privacy and data management. PHO’s ability to develop a centralized program for 34 PHUs was also facilitated by having core requirements for contact management established centrally by the MOH.

Partnerships were established with provincial and federal government agencies to resource and manage the workforce supporting phone calls and data entry tasks. Partner agencies included Health Canada, Ontario Health, and the Department of National Defence, which participated from April 2020 to September 2020, and Statistics Canada, which participated from July 2020 onward. In total, more than 1500 staff from the four government agencies received training to support the CTI. The number of callers involved in the program fluctuated over time as new partnerships were developed and demand for support changed.

### Program scope

The scope of the CTI was based on which tasks could be supported safely and effectively by a non-medical workforce in addition to where there would be value in providing surge capacity support. PHUs would follow up with contacts on their own when they had the capacity or if high-risk contacts did not meet the CTI’s eligibility criteria. PHUs were instructed not to send any contacts who required unique or tailored messaging that was not captured in the CTI standard scripts. As a result of these criteria, PHUs continued to manage a proportion of high-risk close contacts, leverage other workforce supports, or collaborate with other partners (e.g. Indigenous health service providers).

### Data management

The CTI required a comprehensive data management strategy to support information exchange between the COVID-19 workforce supported by government agencies, PHO overseeing data quality and reporting, and the 34 PHUs leading case investigations. In 2020, Ontario implemented a new provincial information system: the Public Health Case and Contact Management Solution (CCM). PHUs used CCM to enter details about COVID-19 cases and high-risk close contacts. PHUs could transfer contacts to the CTI using a custom assign button that would automatically generate follow-up tasks for callers. Following training and the completion of confidentiality and CCM user agreements, callers were given access to CCM. Customized user profiles were set up in CCM to simplify the transfer of information and limit access to information within CCM.

**Fig. 2:**
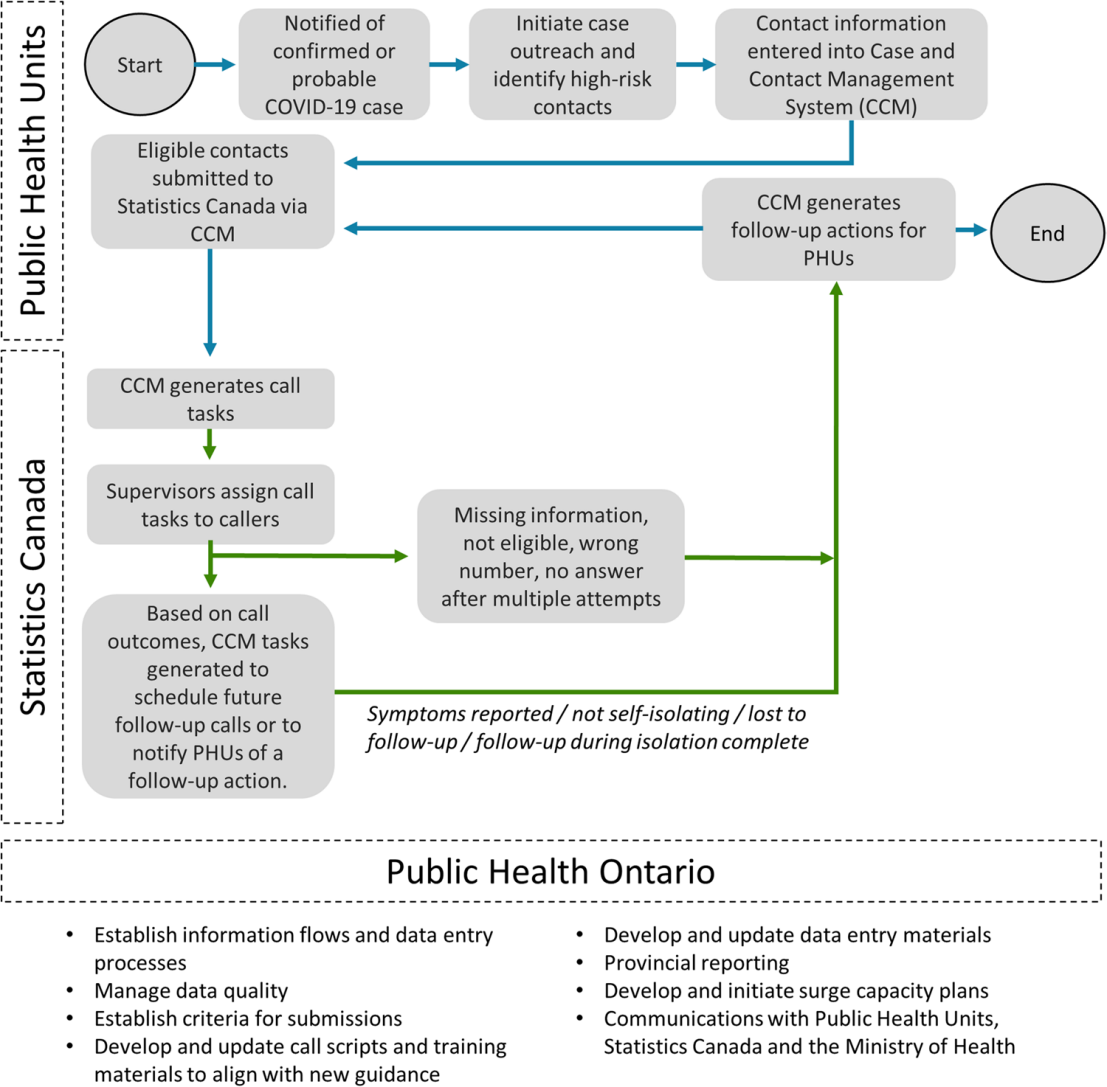
Overview of roles and responsibilities and how information was exchanged between partners supporting Public Health Ontario’s (PHO) Contact Tracing Initiative

Callers could select the appropriate call script and then advance through each page of the guided flow while speaking with the contact. Phone calls involved a symptom assessment and the delivery of information about testing and self-isolation. Selected answers directed the flows in CCM and, ultimately, what guidance was provided to contacts. Figure 2 provides an overview of how data and information were exchanged between PHUs and the partner organizations supporting this initiative.

The transition to CCM started during the early periods of the pandemic in Ontario, with a focus initially on COVID-19 case and contact management and reporting. The CCM provided an essential infrastructure support and also presented significant challenges for public health professionals with the need to quickly learn the new system and adapt existing processes—all while maintaining case and contact management capacity. During the transition period, access to the CTI was interrupted for some PHUs that had not yet transitioned to CCM. When CCM was fully implemented, uptake of the CTI increased as this new system simplified the process of sending contacts to the program.

### Workforce capacity and training requirements

**Fig. 3:**
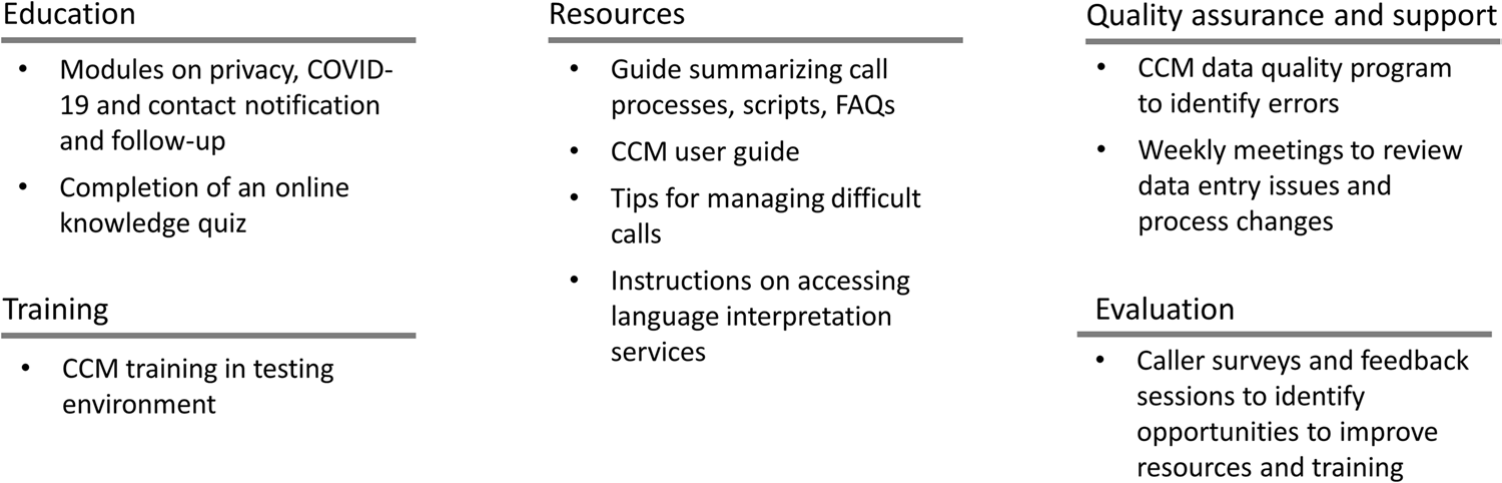
Overview of education, training, and qualitative assurance supports for Public Health Ontario’s (PHO) Contact Tracing Initiative

Figure 3 outlines the supports that were used to prepare callers with respect to education, training, resources, quality assurance, and evaluation. A team at PHO monitored the completion of training requirements and managed a daily data quality program to identify and mitigate data entry issues.

In the case of Statistics Canada, the organization had a large workforce of professional interviewers who were already trained and skilled in following scripts, data entry procedures, and client interactions. Existing processes were already in place to support recruitment and to manage a large workforce. Staff could be cross-trained across regional areas and on the policies of other jurisdictions. This allowed staff to support across jurisdictions as call demands changed and workload fluctuated.

The CTI was designed to support a high volume of calls. This was facilitated by having set criteria for submissions to the program, standardizing scripts, automating flows, and simplifying data entry tasks. An important principle guiding the development and updates to the CTI was to standardize and simplify, where possible, call procedures and data entry requirements.

### Monitoring and evaluation

From the beginning, the PHO team allocated resources to performance monitoring, quality improvement, and evaluation activities. This included:

- Monitoring and reporting on the volume of submissions and the timeliness of contact notification (e.g. proportion of contacts reached within 24 and 48 h of submission to the program).
- Surveying callers and organizing weekly meetings with supervisors to identify opportunities to improve resources, training, and other supports.
- Documentation of milestones and changes to the program.

Early evaluation activities included outlining a logic model for the program which helped focus an evaluation strategy for the CTI. This strategy involved documentation, monitoring uptake, supporting team debriefs to identify opportunities for improvement, and building in opportunities for program stakeholders to reflect on challenges and lessons learned.

## Outcomes

### Program uptake

In the first few months of the program, there were low levels of participation among the 34 PHUs and a lower-than-expected call volume relative to the number of staff trained. However, uptake started to increase shortly following the initial implementation phase with increased awareness of the program and with changes in capacity and demand at the PHU level. The transition to CCM also enabled more PHUs to start using the program.

In the 23 months that the CTI operated, over one million phone calls were made to high-risk contacts in Ontario. Almost all PHUs (*n* = 33/34) accessed support from the CTI to reach high-risk contacts. Additional supports were implemented by the MOH in later periods of the pandemic to increase capacity for the full case and contact management process, including a workforce that could be directly assigned to PHUs if they were managing a high volume of cases and contacts or needed more complex forms of support.

**Fig. 4:**
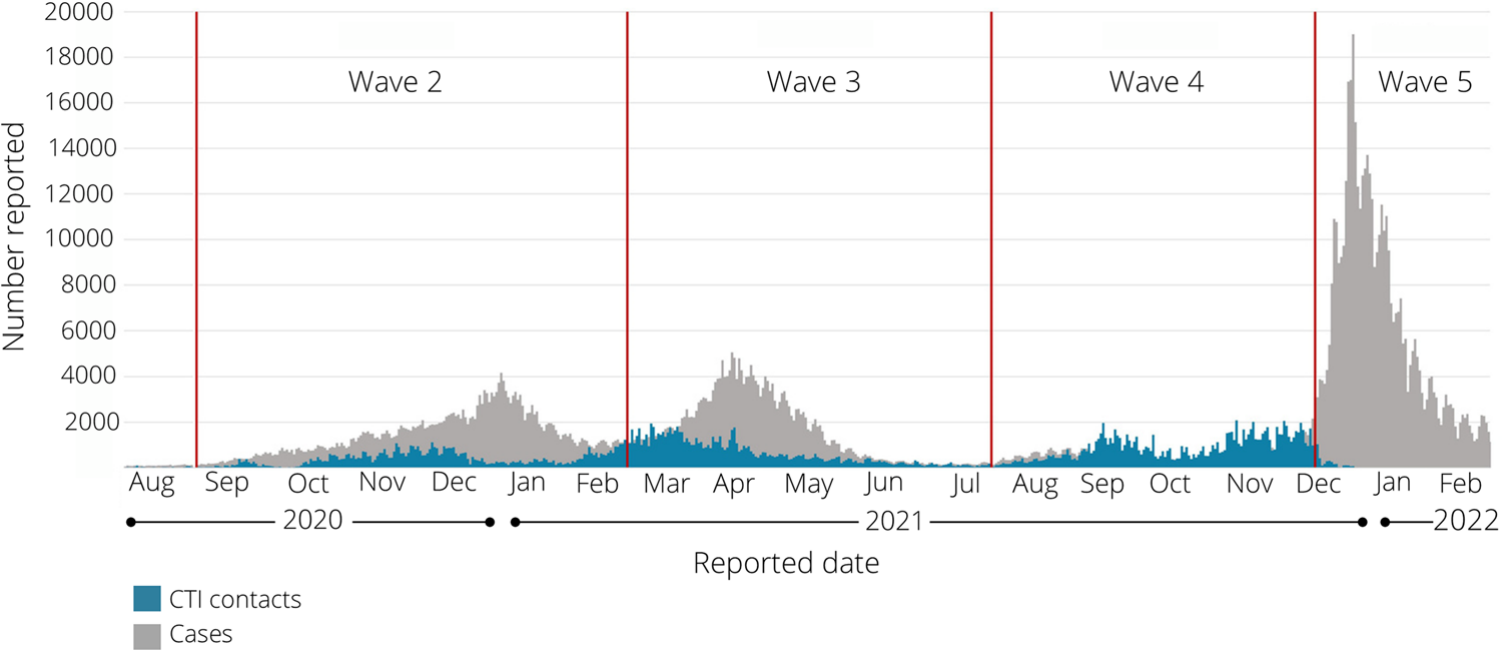
Number of reported cases of COVID-19 in Ontario and number of contacts submitted to Public Health Ontario’s Contact Tracing Initiative (CTI) for follow-up. Data sources: Public Health Ontario’s COVID-19 Data Tool (PHO, 2022) and PHO internal data on the CTI

Figure 4 captures trends in the number of high-risk contacts submitted to the CTI for follow-up along with the number of reported cases of COVID-19 in Ontario. There was variation in the uptake of the program as each PHU experienced different needs at different times during the pandemic. For example, many PHUs relied extensively on the CTI to support contact follow-up for exposures in schools. Periods when schools were closed for in-person learning, along with summer and holiday breaks, had a significant impact on the volume of contacts submitted. Participating PHUs also noted that their use of the CTI was influenced by rising cases of COVID-19 in their community, changes to public health measures, emerging COVID-19 variants of concern, managing additional pandemic priorities (e.g. immunization campaigns), and sudden surges. Most notably, submissions of contacts to the program for follow-up declined each time cases started to peak as PHU capacity for case investigations and data entry was understandably impacted when there were increasingly high case numbers.

Ultimately, the pandemic response in Ontario shifted with the emergence of the Omicron variant in December 2021. At this time, provincial guidance was updated by the MOH to no longer require contact notification outside of a set group of high-risk settings (such as congregate settings). As a result, PHUs no longer required the same volume of contact notification calls to be made on a daily basis, resulting in a sharp decline in program use by the end of December 2021, and a pause to the program operations by late February 2022.

### Program strengths and limitations

A key component of the evaluation of the CTI was to gather evaluative feedback from PHU key informants to capture their perspective on strengths and limitations of the program. In the spring of 2022, the evaluation team reached out to all PHUs that had used the CTI. Online Resource 1 (see Supplementary Material) outlines the questions that PHUs were asked to reflect on. Thirty-one key informants affiliated with 17 PHUs provided feedback. The majority of informants were in a manager or team lead position overseeing case and contact management. Overall, we found that perspectives on strengths and limitations of the CTI were consistent across PHU key informants.

Key informants emphasized that the CTI was able to help manage a high volume of calls to high-risk close contacts and provide timely support to encourage self-isolation. Another advantage was having training and resources come from a centralized source. It would have been resource intensive for each PHU to update materials or to provide training to callers whenever there was a change in guidance.

Another strength was the standardized approach to call scripts. PHU participants shared that it was reassuring to know that all high-risk close contacts were receiving the same information through the program. PHUs also shared that they could use the resources developed by PHO to update their own internal resources quickly, if needed. During the first few months the CTI was operating, there were over 180 requests to adapt and/or use PHO resources and training materials. Last, PHU participants also emphasized the important role the CTI played in alleviating extensive workload pressures on staff. 

The program faced some challenges over its tenure. A common limitation noted by the PHUs was a delay in updates to call scripts each time provincial guidance was updated. The scripts were accessed by callers through the new information system (CCM). Changes to script language could only be made when the team responsible for CCM scheduled a system update. While having scripts embedded in the data management system helped reduce data and information errors and simplified call processes, it took longer to update and implement the scripts compared to having separate standalone documents. As guidance changes became more and more frequent, it became evident that the system needed to be more flexible to respond to these changes quickly. Relatedly, there was a learning curve around implementing a new provincial data entry system in the midst of a pandemic. All users of the system, PHU staff, CTI team at PHO, and the Statistics Canada callers, had to learn to navigate a new data management system and continually adapt as improvements were implemented. 

Another tension felt by users was the balance between designing a standardized approach for a centralized system across the province and the desire to have a program that could be tailored to meet the different needs of PHUs. For example, if a PHU had enhanced guidance for case and contact management that differed from the standardized script, they could not use the program. Some PHUs also noted how it was limiting that the program was not able to support more comprehensive clinical documentation on client interactions or provide support for more complex tasks in the contact management process. As noted earlier, the scope of support provided by the CTI needed to align with the resources available, including access to a workforce that did not have a medical background. 

## Implications

The CTI provided PHUs with a timely surge capacity system which reached a high volume of high-risk close contacts in a short period of time. There were a number of lessons learned that would apply to situations where public health needs to scale case and contact management capacity quickly. One area of learning was around the importance of continuing to assess the fit between the scope and capacity of a centralized workforce and the types of supports needed by PHUs. The CTI was most valuable when PHUs had to manage sudden increases in the volume of high-risk close contacts following changes in public health measures and when managing competing priorities, and became less valuable when there was a need to tailor guidance and offer more complex forms of support. When considering whether this model could be used in the future or by another jurisdiction, it would be important to consider that this program was only able to deliver and collect standardized information (as opposed to tailored health information and guidance).

Embedding scripts and flows in the new CCM system was an innovative feature of the CTI that aimed to reduce information errors. However, this resulted in delays getting call scripts updated following guidance changes. Sharing standalone scripts could be a more nimble option when guidance and messaging need to change quickly and often.

In the early development phase, we found there was a lack of guidance on how to assess the performance and effectiveness of this type of extrinsic surge capacity program. In addition, we found that conceptual frameworks seemed more helpful for understanding surge capacity for healthcare systems as opposed to more upstream public health service delivery (Bonnett et al., 2007). Recently, Vogt et al. (2022) published a scoping review of indicators to monitor contact tracing performance and assess contact tracing systems, still noting gaps in input, outcome, and impact indicators. It would be valuable to extend work in this area to consider overarching components and related goals to guide development, planning, and evaluation drawing on different jurisdictions’ experiences with the rapid development (e.g. Celentano et al., 2021) and maintenance of contract tracing programs.

Lessons learned from this initiative could support future emergency preparedness activities focused on surge capacity support for case and contact management. It would be valuable for our partners in the public health system to examine the resource implications and outcomes of different options that were used to increase capacity for case and contact management in provinces and territories across Canada. There were significant time constraints during the early phases of the COVID-19 response to develop new processes, data management systems, and training to build surge capacity programs for case and contact management. Lessons learned from this surge capacity program demonstrate the importance of preparedness activities that: identify gaps and opportunities to advance baseline training for case investigators and contract tracers; identify opportunities to enhance and test data management systems to ensure they can support a high volume of investigations and support collaboration; and map out potential partnerships that could be leveraged to scale up case and contact management support in the future.

## **Implications for policy and practice**

What are the innovations in this policy or program?

- Public Health Ontario’s Contact Tracing Initiative was an innovative program that:
  - ○ identified contact management activities that could be supported by a non-public health workforce;
  - ○ leveraged existing resources and expertise;
  - ○ forged new partnerships;
  - ○ optimized the use of a new provincial information system;
  - ○ established processes that reduced errors in information sharing and data entry; and
  - ○ developed new training resources that were adapted across Ontario and beyond.
- This model of surge capacity could be useful in the future if public health units needed support with a very high volume of calls and standardized information could be collected and delivered to high-risk contacts.

What are the burning research questions for this innovation?

- Additional research would be valuable to:
  - ○ develop conceptual frameworks that could be used to assess the performance and effectiveness of surge capacity programs specific to case and contact management;
  - ○ understand at what time points during the pandemic was case and contact management most impactful for interrupting the spread of COVID-19 and reducing morbidity and mortality;
  - ○ determine what factors optimized adherence with self-isolation; and
  - ○ better understand the needs of the public in the case investigation and contact management process.

## Supplementary Information

Below is the link to the electronic supplementary material.

Supplementary file1 (DOCX 26 kb)

## Data Availability

Program materials and additional evaluation findings could be available upon reasonable request.

## References

[CR1] Bonnett CJ, Peery BN, Cantrill SV, Pons PT, Haukoos JS (2007). Surge capacity: A proposed conceptual framework. American Journal of Emergency Medicine.

[CR2] Celentano J, Sachdev D, Hirose M, Ernst A, Reid M (2021). Mobilizing a COVID-19 contact tracing workforce at warp speed: A framework for successful program implementation. The American Journal of Tropical Medicine and Hygiene.

[CR3] Ontario Agency for Health Protection and Promotion (Public Health Ontario). (2022). Ontario COVID-19 data tool - COVID-19 daily case counts and rates by episode date in Ontario. [Internet]. Toronto, ON: King’s Printer for Ontario. https://www.publichealthontario.ca/en/data-and-analysis/infectious-disease/covid-19-data-surveillance/covid-19-data-tool?tab=trends. Accessed 15 Sep 2022.

[CR4] Vogt F, Kurup KK, Mussleman P, Habrun C, Crowe M, Woodward A (2022). Contact tracing indicators for COVID-19: Rapid scoping review and conceptual framework. PLoS ONE.

[CR5] World Health Organization. (2021). Contact tracing in the context of COVID-19: Interim guidance, 1 February 2021. World Health Organization. https://apps.who.int/iris/handle/10665/339128. Accessed 9 Nov 2022.

