# Supplementary material for: Lessons learned from implementing a surge capacity support program for COVID-19 contact management in Ontario
Source: Can J Public Health. 2023 May 3;114(4):555–62. doi: 10.17269/s41997-023-00773-6 (PMC10155666; doi:10.17269/s41997-023-00773-6)
Supplement: Supplementary file 1 — Supplementary file1 (DOCX 26 kb) [file 41997_2023_773_MOESM1_ESM.docx]

**Public Health Ontario’s Contact Tracing Initiative: Public Health Unit Interview Guide**

**Background**

1. Can you start by sharing how long you have been overseeing contact management at your health unit during the COVID-19 pandemic?

- Can you share when you started and if you supported that whole time or rotated through this position?

**Uptake**

We’ve had 32 of the 34 Public Health Units use this program at various stages of the pandemic. Use of the Contact Tracing Initiative fluctuated quite a bit. We would like to understand the factors that influenced the use of this surge capacity program, and have two questions about this topic.

1. Based on your experience, what were the driving factors that impacted your health unit’s participation in the Contact Tracing Initiative?

- Prompts: Refer to the data we have about participation in the program. *For example “We noticed that your health unit used the program a lot during the fall of 2021, but not much before then. Do you recall any particular factors that influenced participation at that time?”*
- How did you determine if/how you would participate in the Contact Tracing Initiative? *Did you have any particular signals or triggers?*
- What influenced the proportion of contacts you would send to the Contact Tracing Initiative relative to what was being entered into CCM? *(E.g. if you had 100 contacts that qualified but sent 50, why did that happen?)*

1. Do you recall if your health unit had to **discontinue** contact follow-up all together?
   - [If yes]. Can you share the reasons why your health unit discontinued contact follow-up? *What went into that decision?*

**Feedback on PHO’s Contact Tracing Initiative**

Now we want to ask you a few questions to get feedback on some of the processes specific to the Contact Tracing Initiative. We want to encourage you to feel free to share both strengths and weakness.

1. Can you tell me about your health unit’s experiences **submitting contacts** to the program? Was it clear or were there ever challenges with that process?
2. Next I’m curious if you are aware of the **supporting program resources**? If so, which ones did you use the most or which was most helpful?
   - Prompt: for example the CCM Quick Reference Guide for Public Health Units; the PHO.CCM inbox? Did you ever use the script?
   - What did you like about the resources? What did you not like?
   - Was there anything that would have been useful but was not available?
3. How was your experience **communicating** with PHO about this program in terms of timeliness of response and quality of information provided?
   - *Prompts: Were your inquiries answered promptly and completely? Anything you would suggest we change or consider doing differently? Anything that went particularly well?*
4. Do you have any feedback on the **turnaround time** for contact notification and follow-up for contacts your PHU submitted to CTI? Anything about the time from submission to CTI to the first call; or between first call to follow-up calls?
5. Next, is there anything you would like to share about the **quality of information** delivered to contacts in the CTI?
   - *For example, accuracy of the information in the scripts? Quality of the guidance being given?*
6. And lastly, do you have any feedback on the **quality of data entered** into CCM that went back to the PHU, specifically from the CTI (for example the tasks, the notes on interactions with contacts)?

**Centralized model**

1. For our last official question, we wanted to talk about the workforce support options. The Contact Tracing Initiative used a centralized model where the StatsCan workforce followed a standardized script and process to support all PHUs. Your health unit may also have used other options for case and contact management support (like direct assign options either with StatsCan or the PWF). Based on your public health unit experience and perspective, can you share any advantages or disadvantages of the centralized model used for the Contact Tracing Initiative?

**Wrap-up**

1. We’re getting ready to wrap up and before we do, wanted to check if there is anything else around limitations of the program you would like to share? Or anything useful or helpful about the program that was beneficial for your PHU and that we have not covered yet?
